# Biodistribution of [^11^C]-Metformin and mRNA Expression of Placentae Metformin Transporters in the Pregnant Chinchilla

**DOI:** 10.1155/2019/9787340

**Published:** 2019-04-30

**Authors:** Maria Dahl Overgaard, Christina Søndergaard Duvald, Mikkel Holm Vendelbo, Steen Bønløkke Pedersen, Steen Jakobsen, Aage Kristian Olsen Alstrup, Emmeli Mikkelsen, Per Glud Ovesen, Michael Pedersen

**Affiliations:** ^1^Comparative Medicine Lab, Aarhus University Hospital, Aarhus, Denmark; ^2^Department of Nuclear Medicine and PET Centre, Aarhus University Hospital, Aarhus, Denmark; ^3^Department of Biomedicine, Aarhus University Hospital, Aarhus, Denmark; ^4^Endocrinology and Internal Medicine, Aarhus University Hospital, Aarhus, Denmark; ^5^Department of Obstetrics and Gynecology, Aarhus University Hospital, Aarhus, Denmark

## Abstract

**Background:**

While metformin is the first-line pharmacological treatment of diabetes mellitus type 2, this drug is not considered safe to use in pregnant women because of its unknown consequences for the fetus. In this study, we aimed to investigate the biodistribution of metformin in the pregnant chinchilla, a species exhibiting placental characteristics comparable with the pregnant woman. Furthermore, we aimed to investigate the expression of metformin transporters in humans and chinchillas, respectively, in order to evaluate the pregnant chinchilla as a novel animal model for the use of metformin in pregnancy.

**Methods:**

Three chinchillas in the last part of gestation were injected with [^11^C]-metformin and scanned by PET/CT for 70 minutes to visualize the distribution. To investigate the difference in expression of placenta transporters between humans and chinchillas, PCR was performed on samples from five chinchilla placentae and seven human placentae.

**Results:**

Dynamic PET with [^11^C]-metformin showed that the metformin distribution in chinchillas was similar to that in nonpregnant humans, with signal from kidneys, liver, bladder, and submandibular glands. Conversely, no radioactive signal was observed from the fetuses, and no metformin was accumulated in the chinchilla fetus when measuring the SUV. PCR of placental mRNA showed that the human placentae expressed OCT3, whereas the chinchilla placentae expressed OCT1.

**Conclusion:**

Since metformin did not pass the placenta barrier in the pregnant chinchilla, as it is known to do in humans, we do not suggest the chinchilla as a future animal model of metformin in pregnancies.

## 1. Introduction

Today, 5% of all pregnant women develop gestational diabetes mellitus (GDM), and this prevalence is expected to increase due to the increasing proportion of overweight and obesity [1]. Diabetes in pregnancy can lead to adverse complications for both the mother and the unborn child [2]. Subcutaneous insulin injection is often used to control the blood glucose levels in pregnant women suffering from GDM. The insulin requirement changes throughout pregnancy, and treatment requires regular monitoring. Managing GDM with insulin injections can be challenging, particularly for those who have never used insulin injections before, leading to the risk of poor compliance. Alternatively, it is much easier to prescribe an antidiabetic oral drug. Metformin is first-line pharmacological treatment of diabetes mellitus type 2 (DM2) [3] and polycystic ovary syndrome (PCOS). It has few side effects, mainly gastrointestinal symptoms such as diarrhea and nausea [4], and is associated with a low risk of hypoglycemia and lactate acidosis [5].

Because metformin is a cationic base at physiological pH and has a hydrophilic structure, the passive diffusion through cell membranes is negligible. Instead, transporters must mediate uptake of metformin, both distribution and elimination. Metformin is substrate of different kinds of solute carrier transporters (SLC): organic cation transporters (OCT1, OCT2, and OCT3) and multidrug and toxin extrusion transporters (MATE1 and MATE2) [6]. It has been widely used in pregnancy for more than 40 years [7]. However, the pharmacokinetics of metformin and especially the biodistribution are not fully understood. Metformin passes across the placenta, and studies using umbilical cord blood samples from women treated with metformin during third trimester showed that the fetus was exposed to concentrations approaching those in the maternal circulation [8]. Two recent reviews concluded that metformin had no short-term adverse effects on pregnancy, potential benefits in the neonatal period, but limited long-term follow-up information [7, 9].

In the human, metformin is excreted unchanged in urine. In the fetus, the amniotic fluid is derived from fetal urination, and the amniotic fluid is primarily eliminated through fetal swallowing. The fetal metabolism of metformin is unknown; however, it could be speculated that metformin is excreted to the amniotic fluid, swallowed by the fetus and then reabsorbed to the fetal circulation. Hence, the fetus theoretically could have an increased concentration or accumulation of metformin. Thus, a recent study showed that children aged 5–10 years exposed to metformin in utero have higher body mass indices and measures of central adiposity, and increased risk of obesity [10]. Metformin treatment in pregnancy is a controversial option, and this drug is therefore not used during pregnancy in many countries [7].

**Figure 1:**
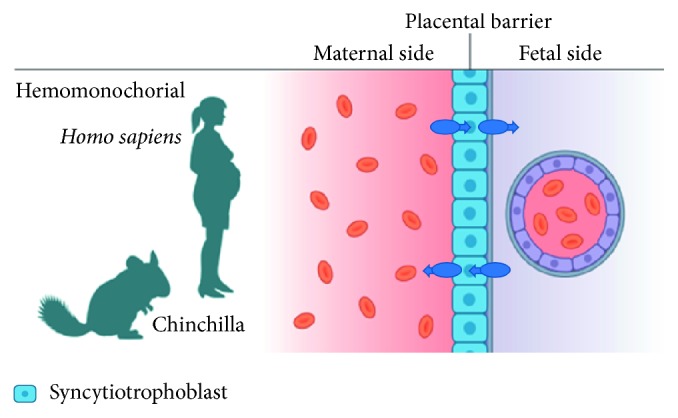
Transport of metformin across the placenta barrier depends on both influx transporters and efflux transporters. Influx and efflux transporters on both sides are necessary for bidirectional transport across the placenta. The placenta barrier of humans and chinchillas is called hemomonochorial because the maternal and fetal blood are divided by only one layer of syncytiotrophoblasts.

For metformin to cross placenta, there must be both influx and efflux transporters in the trophoblast cells (Figure 1) [11]. The exact mechanism of transplacental metformin transport is not known. In order to study metformin during pregnancy, an appropriate animal model is needed. Such animal model must be carefully considered to investigate transplacental transports of substances. Krogh's principle should be applied for every pathological or physiological condition, and the most appropriate animal model must be chosen. An appropriate animal model of pregnancy should account for the number of fetuses, the length of gestation period, and the placental structure. The *Chinchilla lanigera* poses excellent characteristics in this matter, carrying only one or two cubs and having a hemomonochorial placenta barrier like the human placenta (Figure 1) [12]. Furthermore, the chinchilla has a long gestation period (115 days), and the offspring are precocious (matured neurodevelopment at birth, e.g., born with open eyes) like the human newborn [13]. Finally, the relatively large size of the chinchilla fetuses allows for the use of diagnostic imaging methods [12]. These characteristics suggest that the chinchilla is superior to other rodents as animal model of human pregnancy.

The aim of this study was to investigate the biodistribution and pharmacokinetics of metformin in a human translational animal model. We address this aim using a noninvasive approach, employing ^11^C-labeled metformin ([^11^C]-metformin) [14, 15] and positron emission tomography (PET) [15] in the pregnant chinchilla. [^11^C]-metformin has previously been used to study biodistribution in humans but never during pregnancy.

## 2. Materials and Methods

### 2.1. Animal Handling

We included clinically healthy, pregnant chinchillas purchased from a local commercial breeder. They were single housed, fed with a chinchilla pellet diet, and had ad libitum access to tap water. The environmental temperature was around 20°C with 12 hours of light cycles. The animals were fasted 6 hours prior to anesthesia.

### 2.2. PET-CT Examination

Three pregnant chinchillas in the last part of their pregnancy (gestation days 82–93) with a mean weight of 841 g (774–902 g) were used in this study. Two of them were carrying only one fetus and one had two fetuses. Anesthesia was induced with 5% isoflurane in a gas chamber and maintained with a mask delivering 1.5%–3% isoflurane. Respiration was monitored during the scan, and anesthesia protocols were followed by a veterinarian. Prior to the PET examination, intravenous access was gained through the tail vein and used for radiotracer injection. The animals were placed in the field of view in a PET-CT system (Siemens, Erlangen, Germany). ^11^C-metformin was prepared as previously described [15], containing metformin dissolved in aqueous (NH_4_)_2_HPO_4_ (100 mM, pH 5). A bolus of ^11^C-metformin was injected (∼1 min) followed by 0.5–1 mL saline at time = 0. A volume of 3.5–5 mL was injected intravenously with a mean radioactive dosage of 40 MBq (17–86 MBq; specific activity: 30–90 GBq/micromole). The animals underwent 70 min of dynamic PET/CT scan.

List-mode acquired PET data were reconstructed and divided into frames with the frame structure: 12 × 5 s, 5 × 10 s, 2 × 30 s, 4 × 60 s, 4 × 120 s, and 11 × 300 s. Volume of interest (VOI) was defined using PMOD (PMOD Technologies LLC, Zürich, Switzerland) in order to evaluate the tracer activity in specific areas.

### 2.3. Analysis of Metformin mRNA Transporters in Placenta

#### 2.3.1. Chinchilla

Five placentae were dissected from chinchillas at gestation days 82–101. The placentae were rinsed in saline and divided into small parts, and the pieces were snap-frozen in liquid nitrogen. Samples were stored at −80°C until analysis.

#### 2.3.2. Human

Seven placentae were collected immediately after planned cesarean sections from uncomplicated pregnancies. A paracentral grape-sized piece of villous tissue was excised, rinsed in saline, divided into smaller pea-sized pieces, and snap-frozen in liquid nitrogen. Samples were stored at −80°C until analysis.

#### 2.3.3. qPCR Analysis

RNA was extracted using TRIzol (Gibco BRL; Life Technologies, Roskilde, Denmark). RNA was quantified using a NanoDrop 8000 spectrophotometer (Thermo Scientific, Waltham, MA). Integrity of the RNA was checked by visual inspection of the two ribosomal RNAs on an agarose gel. cDNA was synthesized using a Verso cDNA kit (Ab-1453; Thermo Scientific) with random hexamer primers. The PCR reactions were performed in duplicate using LightCycler SYBR Green master mix (Roche Applied Science, Indianapolis, IN) in a LightCycler 480 (Roche Applied Science) using the following protocol: one step at 95°C for 3 min, then 95°C for 10 s, 60°C for 20 s, and 72°C for 10 s, and finally, a melting curve analysis was performed. The increase in fluorescence was measured in real-time during the extension step. The relative gene expression was estimated using the “Advanced Relative Quantification” mode of the software version LCS 480 1.5.1.62 (Roche Applied Science), and specificity of the amplification was checked by melting temperature analysis. The following primer pairs were designed using QuantPrime [16]:

  Chinchilla primers:

Housekeeping: beta-2 microglobulin, TGGTGCATGGCGCCTTTATC GACAGTGTGACGTGTGAAACGC product length 70 bp
CH_MATE1 AAGGAGCTGTTGGAGTCAACCC TAACGATGCTGAAGCGCACAGG product length 74 bp
CH_OCT1 GCTGGGCATATAGCTCAGTGGTAG GATTGAACCAAGGGCCTTCAGC product length 62 bp
CH_OCT2 ATTCCCAGCCGCCTTCATTGTC TGACACAGCCCAAGGATAACGG product length 70 bp
CH_OCT3 TTCAGGCCCAGACATCTGAAGG TCTGCTTGGCTCCTGGTAAAGC product length 62 bp

  Human primers:

Housekeeping: beta-2 microglobulin: GAGGCTATCCAGCGTACTCC- AATGTCGGATGGATGAAACCC product length 111 bp
H.MATE1 TCGGCTTATCTTCTGCCTGT CTGGGTAAGCCTGGACACAT product length 197 bp
H.OCT1 TAATGGACCACATCGCTCAA AGCCCCTGATAGAGCACAGA product length 190 bp
H.OCT2 ATGCCCACCACCGTGGACGAT AGGAAGACGATGCCCACGTA product length 128 bp
H.OCT3 (SLC22A3) GGAGTTTCGCTCTGTTCAGG GGAATGTGGACTGCCAAGTT product length 216 bp

All primers were purchased from the same manufacturer (DNA Technology, Risskov, Denmark). A similar setup was used for negative controls, except that the reverse transcriptase was omitted, and no PCR products were detected under these conditions.

### 2.4. Ethical Aspects

The study followed the guidelines for use and care of laboratory animals and was approved by the Danish Inspectorate of Animal Experiments. During anesthesia, the animals were euthanized by an overdose injection of pentobarbital. For the human placentae, the mothers all gave informed consent prior to the placenta harvesting.

## 3. Results

### 3.1. PET

**Figure 2:**
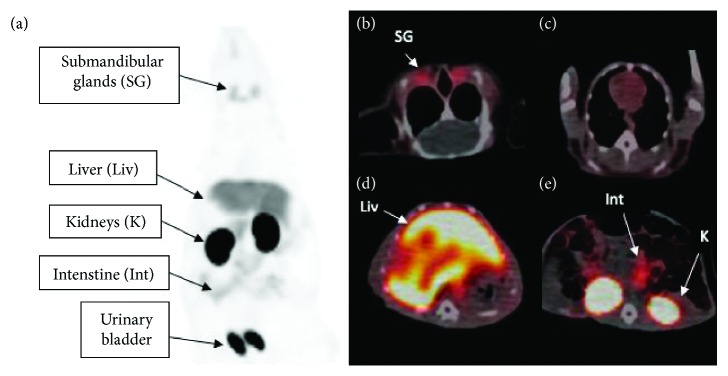
Whole-body [^11^C]-metformin PET scans were performed after i.v. tracer injection. (a) Attenuation corrected maximum intensity projection of [^11^C]-metformin biodistribution in a chinchilla 30 min after injection demonstrates uptake in submandibular glands, liver, kidneys, intestine, and urinary bladder (indicated by arrows). Transaxial slices of [^11^C]-metformin biodistribution on merged PET/CT images (arrows indicate metformin avid organs) are displayed in (b) submandibular glands, (c) thorax, (d) liver, and (e) kidneys.

**Figure 3 fig3:**
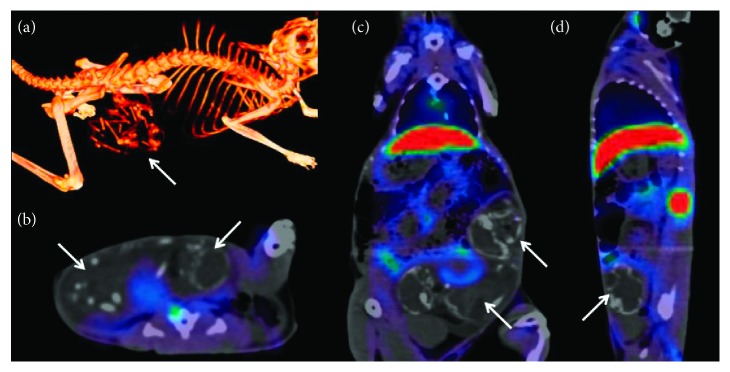
(a) Two fetuses identified in a pregnant chinchilla (the arrow indicates the fetuses) with 3D volume rendering of bone reconstructed CT scan. A summarized [^11^C]-metformin PET/CT scan is visualized in transaxial (b), coronal (c), and sagittal plane (d) (arrows indicate the two fetuses), demonstrating no metformin uptake in the fetuses.

**Figure 4 fig4:**
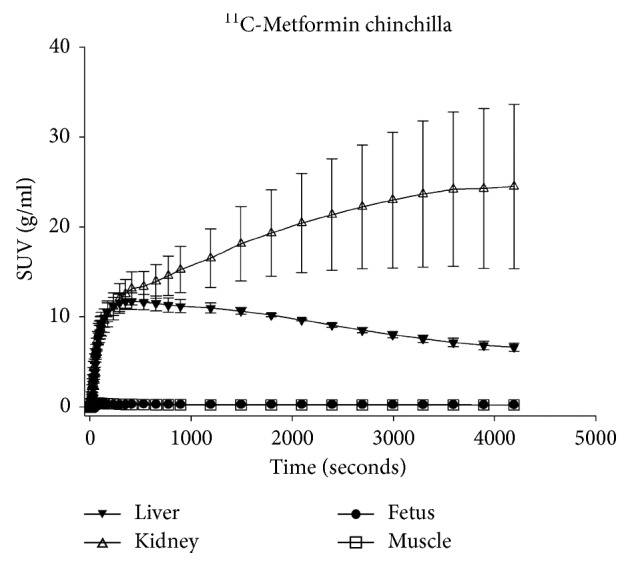
Dynamic PET metformin kinetics. Standardized uptake value (SUV, g/ml) of [^11^C]-metformin in the liver, kidney, fetus, and muscle.

Visual inspection of the PET scans revealed that [^11^C]-metformin was primarily found in the kidneys, liver and bladder, and a small amount in the submandibular glands (Figure 2). Dynamic PET with [^11^C]-metformin showed a high signal from the liver and kidney, but there was no signal from the fetuses (Figures 3(b)–3(d)). Measurements of standardized uptake value (SUV) (Figure 4) showed that radioactive uptake in the liver increased just after [^11^C]-metformin administration and steadily decreased from *t* = 250 s. A relatively high and prolonged uptake was observed in the kidneys. No [^11^C]-metformin was accumulated in the fetus.

### 3.2. Analysis of mRNA for Metformin Transporters in Human and Chinchilla Placentas

**Figure 5:**
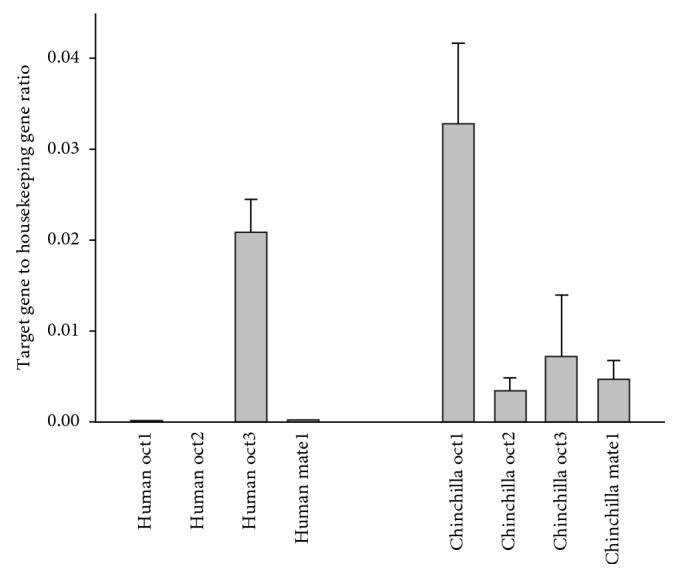
mRNA expression of the metformin transporters in chinchilla and human placentae related to housekeeping gene.

mRNA expression of metformin transporters revealed that human placentae primarily expressed OCT3, whereas chinchillas primarily expressed OCT1 (Figure 5). The expression of other transporters was found very low.

## 4. Discussion

The biodistribution of [^11^C]-metformin has previously been studied in pigs, rats, and humans, but none of them during pregnancy [15]. As the chemical production of [^11^C]-metformin varied from time to time, and because the isotope has a short half-life of around 20 min, the animals included in this study received different volumes (3.5–5 mL) and radioactive dosages (17–86 MBq) of [^11^C]-metformin. However, it was easy to follow the tracer in all PET scans with a signal-to-noise ratio sufficient for subsequent data analyses. The acquired PET images of the pregnant chinchillas revealed a maternal biodistribution of metformin that was comparable to that of nonpregnant humans [14]. The kidney curve (Figure 4) showed a high SUV variation compared to the other curves. However, an interindividual variation is also observed in humans due to both genetic variations and glomerular filtration rate [17]. The accumulation of metformin in the bladder indicated that metformin was excreted through the kidneys similar to humans (Figure 2(a)). We also found a very low SUV in the muscles which is comparable to humans. No uptake of metformin was observed in the chinchilla fetuses. Metformin is normally ingested orally but was administered intravenously in this study. However, as metformin is not metabolized in the body and is excreted uncharged, the mode of drug administration should not influence the distribution.

In humans, metformin transfers from the maternal to the fetal circulation, demonstrated by findings of metformin in umbilical cord blood samples in women taking metformin prior to birth [8]. The exact mechanism how metformin crosses the placenta in humans is not fully elucidated [18]. Nevertheless, the difference in the placental transport and pharmacokinetics of metformin between chinchilla and human suggests some limitations in the use of pregnant chinchillas as an animal model for human pregnancy. An obvious reason why metformin is not found in the chinchilla fetuses could be due to transporters in the trophoblasts. We found that human placentae express OCT3 whereas chinchillas express OCT1 (Figure 5). This difference in transporters could be the reason for the missing uptake of metformin in the chinchilla placenta and fetus. In humans, OCT1 is mainly found in hepatocytes and sometimes referred to as the “liver-specific” OCT. Some placenta studies have also found expression of OCT1 in the human placenta, conflicting our findings [11]. OCT3 is the most abundantly expressed SLC transporter in the placenta in many species, including humans, rats, and mice [11], and it has been referred to as the “placenta specific” OCT. However, the tissue distribution of OCT3 is much broader and thus not specific for placenta [11]. Pregnancy is a dynamic process, and the placenta undergoes adaptations and physiological changes accordingly. In pregnant rats, the expressions of OCT3 and MATE1 change during the different stages of pregnancy [19]. Expression of MATE1 was found in human placenta from first trimester [19]. In the term human placentae, we found no expression of MATE1, confirming previous findings [11].

Although PCR is a valuable technique, PCR is capable only in showing whether mRNA is present, but it is incapable of revealing actual protein expressions. In addition, PCR is unable to identify where the transporters reside inside the trophoblasts. Functional studies in human placentae have found that OCT3 is located in the fetal facing membrane of the placenta [20]. Immunohistochemistry in rat placentae was used to identify OCT3 at the fetal side, like in human placentae, whereas MATE1 was located at the maternal side of the rat placenta [21]. In a study where a human *ex vivo* dual perfusion placenta model was used to study metformin, it was found that the fetal-to-maternal transfer of metformin was significantly higher than the maternal-to-fetal transfer [18]. In theory, metformin could be transported into the trophoblasts and quickly transported out again by efflux transporters and never reach the fetal compartment. A study in rats showed that MATE1 was responsible for the efflux of metformin from the trophoblasts [22], and as both OCT3 and MATE1 are found in the rat placentae, synchronized activity of these transporters is suggested as the transplacental pathway [21]. In mice, OCT3, but not MATE1, is found in the placentae, similar with human placentae from late pregnancy [11]. In terms of placentae metformin transporters, mice appear as a more comparable animal model for studying metformin transport in late pregnancy.

## 5. Conclusion

In conclusion, we found that PET and [^11^C]-metformin administration can be used to study the biodistribution of metformin in a chinchilla animal model, and we found that the biodistribution was comparable to findings previously shown in nonpregnant humans. In chinchillas, however, [^11^C]-metformin did not pass the placenta like in humans. Therefore, we studied the possible differences in metformin transporters between human and chinchilla placenta based on mRNA expression of these transport proteins. We interestingly found that the human placentae mainly expressed OCT3, whereas chinchilla placentae had high expression of OCT1. Based on these results, the *in vivo* pharmacokinetics and biodistribution of metformin in chinchillas do not advocate this animal model as an optimal choice for investigations of metformin in human pregnancy.

## Data Availability

The PET/CT and mRNA data used to support the findings of this study are included within the article in Figures 2 [Fig fig3] [Fig fig4]–5.
